# The need for a cancer open-data platform in Peru

**DOI:** 10.17843/rpmesp.2023.402.12778

**Published:** 2023-06-19

**Authors:** Patricia Socualaya-Sotomayor, Aramis Rafael-Carranza, Yasmina Solar-Benites

**Affiliations:** 1 Public Health Evidence Generation and Analysis Unit (UNAGESP), Instituto Nacional de Salud, Lima, Perú. Public Health Evidence Generation and Analysis Unit (UNAGESP) Instituto Nacional de Salud Lima Peru; 2 Clinical Epidemiology Unit, Universidad Peruana Cayetano Heredia, Lima, Perú. Universidad Peruana Cayetano Heredia Clinical Epidemiology Unit Universidad Peruana Cayetano Heredia Lima Peru; 3 Universidad Nacional Mayor de San Marcos, Lima, Peru. Universidad Nacional Mayor de San Marcos Universidad Nacional Mayor de San Marcos Lima Peru; 4 Hospital Nacional Dos de Mayo, Lima, Peru. Hospital Nacional Dos de Mayo Lima Peru; 5 Hospital Nacional Docente Madre Niño San Bartolomé, Lima, Peru. Hospital Nacional Docente Madre Niño San Bartolomé Lima Peru

To the editor. Cancer is a health priority, thus, having tools to assess its impact on society in clinical, social and economic terms is highly important. These tools should be created within the framework of the National Cancer Law ^1^ and the Digital Government Law, the latter approved with Legislative Decree 1412 that resulted in the creation of a National Open Data Platform, with the aim of facilitating free access to relevant information and its processing, storage, communication and interpretation ^2^. There are several information categories in the aforementioned platform. The health category should include a section containing, in a specific and unified manner, information related to cancer, in order to provide an overview of the population with cancer and the public health situation in Peru, thus facilitating the access to evidence for health decision-making.

Along these lines, we searched the web pages of public health and autonomous entities (such as health professional associations), using the National Comprehensive Cancer Care Plan 2020-2024 ^3^ as reference. The information was categorized into five dimensions: epidemiology, information on pharmaceutical products, coverage, specialized service and specialized care. We present a list of 21 cancer-related sources (Table 1), which were examined in order to verify the availability or non-availability of the data.

**Table 1:** Sources of information on cancer in Peru.

	Source	Type of information	Link
Epidemiology	CDC-Peru (National Center for Epidemiology, Disease Prevention and Control)	Hospital registry of cancer in children and adolescents, no data available.	https://www.dge.gob.pe/portalnuevo/vigilancia-epidemiologica/vigilancia-de-cancer/
		ASIS as of 2018.	https://www.dge.gob.pe/portalnuevo/publicaciones/analisis-de-situacion-de-salud-asis/
		2019-2022 cancer situation report shows limited data.	https://www.dge.gob.pe/portalnuevo/publicaciones/salas-de-situacion-semanal/
	INEN (National Institute of Neoplastic Diseases)	Epidemiological data (as of 2019) and cancer registry in Metropolitan Lima (as of 2015).	https://portal.inen.sld.pe/
	IREN-North (Regional Institute of Neoplastic Diseases - North)	Hospital cancer registry and hospital management data, as of 2021.	https://www.irennorte.gob.pe/index.php
	IREN-South (Regional Institute of Neoplastic Diseases - South)	No access.	http://www.irensur.gob.pe/
	IREN-Center (Regional Institute of Neoplastic Diseases - Center)	With no epidemiological data.	https://portal.irencentro.gob.pe/
	National Death Reporting System - SINADEF (Information in the National Health Information Repository)	Peruvian national and regional mortality rates 2000-2021. Top 20 national causes of death 2000-2021.	http://www.minsa.gob.pe/reunis/data/tasas_mortalidad.asp
	REUNIS (National Health Information Repository)	Percentage of cancer deaths before age 70 (2019).	https://ncdportal.org/CountryProfile/GHE110/Peru https://ncdportal.org/
Pharmaceutical product information	DIGEMID (General Directorate of Medicines, Supplies and Drugs)	Consultation of Sanitary Product Registration; not specific for POP.	https://www.digemid.minsa.gob.pe/rsProductosFarmaceuticos/
		Drug product datasheets (including biosimilars); not specific for POP.	https://www.digemid.minsa.gob.pe/webDigemid/fichas-tecnicas-de-productos-biologicos/
		Price observatory; not specific to POP.	https://opm-digemid.minsa.gob.pe/#/consulta-producto
		Availability of Pharmaceutical Products by DISAS/DIRESAS/GERESAS (12-month analysis) - includes Specialized Warehouses and Institutional Pharmacies; not specific for POP.	https://www.digemid.minsa.gob.pe/webDigemid/publicaciones/disponibilidad-de-productos-farmaceuticos/
	CENARES (National Center for Strategic Resource Sourcing)	Distribution of pharmaceuticals; not specific for POP.	https://intranet.cenares.gob.pe/cenares/abastecimiento/abastecimiento/Default.aspx
	SISMED (Integrated Supply System for Pharmaceuticals, Medical Devices and Health Care Products)	Consumption and price of pharmaceutical products of all pharmacy services of MINSA and regional government facilities; not specific for POP.	https://appsalud.minsa.gob.pe/portal_sismed/?op=56#
	RENETSA (National Network for Health Technology Assessment)	Health Technology Assessment of high-cost oncological pharmaceutical products developed by INS, DIGEMID and EsSalud.	https://www.gob.pe/institucion/ins/colecciones/11902-renetsa
Public IAFAS coverage	SIS (Comprehensive Health Insurance)	Budget Program and Performance Indicators, for Cancer Prevention and Control.	http://www.minsa.gob.pe/presupuestales/?pg=6#contact
	FISSAL (Intangible Solidarity Health Fund)	Funds the diagnosis and treatment of the 7 types of cancer.	https://www.gob.pe/fissal
	EsSalud (Social Health Insurance)	Funder of benefits to EsSalud insured members.	No specific link for cancer.
	SALUDPOL (Health Insurance Fund of the National Police of Peru)	Funder of health benefits for PNP personnel and their families. It has oncology coverage.	https://cdn.www.gob.pe/uploads/document/file/1008207/125-2018-IN-SALUDPOL-GG20200713-20664-1hpenzm.pdf
	FOSPEME (Health Fund for the Army Military Personnel)	Has ONCOEP, a supplemental plan to fund cancer care for Army personnel and their families.	https://iafasep.gob.pe/plan-tu-salud/afiliate-oncoep
	FOSMAR (Navy Health Insurance Fund Administration Institution)	It has a complementary health plan that includes the treatment of oncological diseases (ONCONAVAL).	https://iafasfosmar.pe/examen-preventivo-oncologico/ https://iafasfosmar.pe/planes/plan-onconaval/
Specialized health service	SUSALUD (National Superintendence of Health)	National Registry of Health Service Provider Institutions (RENIPRESS). Hospitalization, chemotherapy and radiotherapy centers.	http://app20.susalud.gob.pe:8080/registro-renipress-webapp/listadoEstablecimientosRegistrados.htm?action=mostrarBuscar#no-back-button
Specialized care	CMP (Medical Association of Peru)	National Registry of Oncology Specialists.	https://www.cmp.org.pe/conoce-a-tu-medico/
	SPOM (Peruvian Society of Medical Oncology)	List of specialist associate members as of 2021.	https://www.spomedica.org/miembros-activos/
	CEP (Peruvian Nurses Association)	Not specific to oncology specialty registration.	https://www.cep.org.pe/validar/

ASIS: Health Situation Analysis, POP: Oncological Pharmaceutical Products, IAFAS: Health Insurance Fund Management Institutions, DISAS: Health Directorates, DIRESAS: Regional Health Directorates, GERESAS: Regional Health Managements, INS: National Health Institute, PNP: National Police of Peru, ONCOEP: Oncology Program of the Peruvian Army, ONCONAVAL: Oncology Program of the Peruvian Navy.

Search was carried out in June 2023

It should be noted that, although efforts have been made to record data on cancer, this information has been published by different sources. We found that 48% (n=10) of the sources were specific for cancer, 24% (n=5) had outdated information and 14% (n=3) were not specific for cancer. In addition, three sources didn’t have information available (two were not linked to the information and one did not publish cancer-related information).

Cancer-related information has been centralized by other countries in different ways, such as the Global Cancer Observatory-GCO (GloboCAN) ^4^, the National Cancer Observatory of the Government of Colombia ^5^ and an Oncology Observatory in Brazil ^6^. These observatories conduct research, create monitoring indicators, identify differences between regions, and recognize gaps in information, among other activities. In Peru, the creation of an observatory has been considered in the Regulations of the National Cancer Law ^7^ and in the Regulations of the Medical Emergency Law for the Timely Detection and Comprehensive Care of Child and Adolescent Cancer ^8^. This observatory could monitor the progress of outcome indicators and processes related to reducing cancer morbimortality in children and adolescents. However, the observatory is still being implemented, despite the fact that Peru is the first country in the region to be considered for the global initiative against childhood cancer ^9^, and having announced the creation of a National Observatory of Childhood Cancer ^10^. In view of the above, we consider that the sources of information in Peru could be used to create an open data platform on cancer, which would facilitate free access to information. The National Center for Epidemiology, Prevention and Disease Control (CDC) Peru, along with the Directorate for Cancer Prevention and Control could manage the platform.

In conclusion, creating a Cancer Open-Data Platform is possible and advisable. It could be created by unifying different sources of information, inter-institutional collaboration, as well as the economic sustainability and optimization of the sources of information. This platform could facilitate monitoring the epidemiological, economic, and health care aspects as well as other factors related to the cancer situation in Peru. This initiative could generate organized, focused and representative information on cancer in Peru, which could contribute to comprehensive, free and dignified care for the population.
