# Supplementary material for: The need for a cancer open-data platform in Peru
Source: Rev Peru Med Exp Salud Publica. 2023 Jun 19;40(2):247–9. doi: 10.17843/rpmesp.2023.402.12778 (PMC10953640; doi:10.17843/rpmesp.2023.402.12778)
Supplement: Translation

# Translation: Necesidad de una plataforma de datos abiertos de cáncer en el Perú

**Authors:** Patricia Socualaya-Sotomayor, Aramis Rafael-Carranza, Yasmina Solar-Benites

**Affiliations:** 1 Unidad de Análisis y Generación de Evidencias en Salud Pública (UNAGESP), Instituto Nacional de Salud, Lima, Perú.; 2 Unidad de Epidemiología Clínica, Universidad Peruana Cayetano Heredia, Lima, Perú.; 3 Universidad Nacional Mayor de San Marcos, Lima, Perú.; 4 Hospital Nacional Dos de Mayo, Lima, Perú.; 5 Hospital Nacional Docente Madre Niño San Bartolomé, Lima, Perú.

Sr. Editor. El cáncer es una prioridad sanitaria, por lo que es importante contar con herramientas para evaluar su impacto en la sociedad en términos clínicos, sociales y económicos. En el marco de la Ley Nacional del Cáncer ^1^ y de la Ley de Gobierno Digital, esta última aprobada con Decreto Legislativo 1412 que dio lugar a la creación de una Plataforma Nacional de Datos Abiertos, con la finalidad de facilitar el acceso libre a la información relevante para su procesamiento, almacenamiento, comunicación e interpretación ^2^. En la mencionada plataforma, existen diversas categorías de información. Es necesario que, dentro de la categoría denominada Salud, se pueda incluir una sección que brinde, de manera específica y unificada, información relacionada al cáncer, con el fin de alcanzar una visión de la salud pública y de la población afectada de cáncer en el país, y de esta manera contribuir al acceso de la evidencia para la toma de decisiones en salud.

En esta línea, se realizó una búsqueda en las páginas web de las entidades públicas de salud y entidades autónomas (como los colegios profesionales de la salud), y se tomó como referencia al Plan Nacional de Cuidados Integrales del Cáncer 2020-2024 ^3^. La información hallada se categorizó en cinco dimensiones: epidemiología, información de productos farmacéuticos, cobertura prestacional, servicio especializado y atención especializada. A continuación, presentamos el listado de 21 fuentes de información relacionadas con cáncer (Tabla 1), las cuales fueron examinadas para verificar la disponibilidad o no de los datos, según corresponda.

**Tabla 1:** Fuentes de información sobre cáncer en el Perú.

	Fuente de información	Tipo de información	Enlace
Epidemiología	CDC-Perú (Centro Nacional de Epidemiología, Prevención y Control de Enfermedades)	Registro hospitalario de cáncer en niños y adolescentes, sin datos.	https://www.dge.gob.pe/portalnuevo/vigilancia-epidemiologica/vigilancia-de-cancer/
		El ASIS es al 2018.	https://www.dge.gob.pe/portalnuevo/publicaciones/analisis-de-situacion-de-salud-asis/
		La sala situacional de cáncer 2019-2022 muestra datos limitados.	https://www.dge.gob.pe/portalnuevo/publicaciones/salas-de-situacion-semanal/
	INEN (Instituto Nacional de Enfermedades Neoplásicas)	Datos epidemiológicos (al 2019) y registro de cáncer en Lima Metropolitana (al 2015).	https://portal.inen.sld.pe/
	IREN-Norte (Instituto Regional de Enfermedades Neoplásicas - Norte)	Registro hospitalario del cáncer y datos de gestión hospitalaria, al 2021.	https://www.irennorte.gob.pe/index.php
	IREN-Sur (Instituto Regional de Enfermedades Neoplásicas - Sur)	Sin acceso.	http://www.irensur.gob.pe/
	IREN-Centro (Instituto Regional de Enfermedades Neoplásicas - Centro)	Sin datos epidemiológicos.	https://portal.irencentro.gob.pe/
	Sistema Informático Nacional de Defunciones - SINADEF (Información en el Repositorio Único Nacional de Información en Salud)	Tasas de mortalidad nacional y por regiones del Perú del 2000-2021. 20 primeras causas de muerte nacionales del 2000-2021.	http://www.minsa.gob.pe/reunis/data/tasas_mortalidad.asp
	REUNIS (Repositorio único nacional de información en salud)	Porcentaje de muertes por cáncer antes de los 70 años (2019).	https://ncdportal.org/CountryProfile/GHE110/Peru https://ncdportal.org/
Información de productos farmacéuticos	DIGEMID (Dirección General de Medicamentos, Insumos y Drogas)	Consulta de Registro Sanitario de Productos, no específica para PFO.	https://www.digemid.minsa.gob.pe/rsProductosFarmaceuticos/
		Fichas técnicas de productos farmacéuticos (incluido los biosimilares), no específica para PFO.	https://www.digemid.minsa.gob.pe/webDigemid/fichas-tecnicas-de-productos-biologicos/
		Observatorio de precios, no específica para PFO.	https://opm-digemid.minsa.gob.pe/#/consulta-producto
		Disponibilidad de productos farmacéuticos por DISAS/DIRESAS/GERESAS (análisis a 12 meses), incluye almacenes especializados y farmacias institucionales; no específica para PFO.	https://www.digemid.minsa.gob.pe/webDigemid/publicaciones/disponibilidad-de-productos-farmaceuticos/
	CENARES (Centro Nacional de Abastecimiento de Recursos Estratégicos)	Distribución de productos farmacéuticos, no específico para PFO.	https://intranet.cenares.gob.pe/cenares/abastecimiento/abastecimiento/Default.aspx
	SISMED (Sistema Integrado de Suministro de Productos Farmacéuticos, Dispositivos Médicos y Productos Sanitarios)	Consumo y precio de productos farmacéuticos de todos los servicios de farmacia de establecimientos del Ministerio de Salud (MINSA) y gobiernos regionales, no específicos para PFO.	https://appsalud.minsa.gob.pe/portal_sismed/?op=56#
	RENETSA (Red Nacional de Evaluación de Tecnologías Sanitarias)	Evaluación de tecnologías sanitarias de productos farmacéuticos oncológicos de alto costo, realizados por el INS, DIGEMID y EsSalud.	https://www.gob.pe/institucion/ins/colecciones/11902-renetsa
Cobertura prestacional de IAFAS Públicas	SIS (Seguro Integral de Salud)	Programa presupuestal e indicadores de desempeño, para la prevención y control del cáncer.	http://www.minsa.gob.pe/presupuestales/?pg=6#contact
	FISSAL (Fondo Intangible Solidario de Salud)	Financia el diagnóstico y tratamiento de los siete tipos de cáncer.	https://www.gob.pe/fissal
	EsSalud (Seguro Social de Salud)	Financiador de las prestaciones a los asegurados de EsSalud.	Sin enlace específico para cáncer
	SALUDPOL (Fondo de Aseguramiento en Salud de la Policía Nacional del Perú)	Financiador de prestaciones de salud dirigidas al personal de la PNP y sus familiares. Cuenta con cobertura oncológica.	https://cdn.www.gob.pe/uploads/document/file/1008207/125-2018-IN-SALUDPOL-GG20200713-20664-1hpenzm.pdf
	FOSPEME (Fondo de Salud para el Personal Militar del Ejercito)	Cuenta con ONCOEP, un plan complementario para financiar la atención oncológica del personal del ejército y sus familiares.	https://iafasep.gob.pe/plan-tu-salud/afiliate-oncoep
	FOSMAR (Institución Administradora de Fondo de Aseguramiento en Salud de la Marina de Guerra)	Cuenta con un plan complementario de salud que comprende los tratamientos de enfermedades oncológicas (ONCONAVAL).	https://iafasfosmar.pe/examen-preventivo-oncologico/ https://iafasfosmar.pe/planes/plan-onconaval/
Servicio de salud especializado	SUSALUD (Superintendencia Nacional de Salud)	Registro Nacional de Instituciones Prestadoras de Servicios de Salud (RENIPRESS). Hospitalización, centros quimioterápicos y radioterapia.	http://app20.susalud.gob.pe:8080/registro-renipress-webapp/listadoEstablecimientosRegistrados.htm?action=mostrarBuscar#no-back-button
Atención especializada	CMP (Colegio Médico del Perú)	Registro nacional de especialistas en oncología.	https://www.cmp.org.pe/conoce-a-tu-medico/
	SPOM (Sociedad Peruana de Oncología Médica)	Lista de miembros especialistas asociados al 2021.	https://www.spomedica.org/miembros-activos/
	CEP (Colegio de Enfermeros del Perú)	No es específico para el registro de especialidad en oncología.	https://www.cep.org.pe/validar/

ASIS: Análisis de Situación de Salud, PFO: Productos Farmacéuticos Oncológicos, IAFAS: Instituciones Administradoras de Fondos de Aseguramiento en Salud, DISAS: Direcciones de Salud, DIRESAS: Direcciones Regionales de Salud, GERESAS: Gerencias Regionales de Salud, INS: Instituto Nacional de Salud, PNP: Policía Nacional del Perú, ONCOEP: Programa Oncológico del Ejército del Perú, ONCONAVAL: Programa Oncológico de la Marina de Guerra del Perú.

Búsqueda realizada en junio del 2023.

Cabe resaltar que, si bien existen esfuerzos en el registro de datos sobre cáncer, esta se encuentra publicada en diferentes fuentes. Encontramos que el 48% (n=10) eran específicas para cáncer, el 24% (n=5) correspondía a información desactualizada y en el 14% (n=3) no existía una información específica sobre el cáncer. Además, se hallaron tres fuentes sin disponibilidad de información (dos no contaban con los enlaces correspondientes y uno no publicó la información relacionada con el cáncer).

Existen experiencias de centralización de la información relacionada con cáncer en otros países, como la del Global Cáncer Observatory-GCO (GloboCAN) ^4^, el Observatorio Nacional de Cáncer del Gobierno de Colombia ^5^ y un Observatorio de Oncología en Brasil ^6^. Estos observatorios han permitido realizar investigaciones, crear indicadores de monitoreo, identificar diferencias de los avances entre las regiones, reconocer vacíos en la información, entre otros. En el Perú, se ha considerado la creación de un observatorio en el Reglamento de la Ley Nacional de Cáncer ^7^ y en el Reglamento de la Ley de Urgencia Médica para la Detección Oportuna y Atención Integral del Cáncer del Niño y el Adolescente ^8^. El objetivo de este observatorio sería monitorear el avance de indicadores y procesos relacionados con disminuir la morbimortalidad por cáncer en niños y adolescentes. Sin embargo, esto continúa en proceso de implementación, a pesar de que el Perú es el primer país de la región considerado para la iniciativa mundial contra el cáncer infantil ^9^, con un anuncio para la creación del Observatorio Nacional de Cáncer Infantil ^10^. Por todo lo descrito, consideramos que, en el Perú, existen fuentes de información disponibles con las que se pueda crear una plataforma de datos abiertos de cáncer, que facilite el acceso libre a la información para la población interesada. Esta plataforma podría estar a cargo del Centro Nacional de Epidemiología, Prevención y Control de Enfermedades (CDC) Perú, en coordinación con la Dirección de Prevención y Control del Cáncer.

En conclusión, la creación de una Plataforma de Datos Abiertos de Cáncer es posible y recomendable, mediante la unificación de las diferentes fuentes de información, el trabajo colaborativo interinstitucional, así como la sostenibilidad económica y la optimización del funcionamiento de las fuentes de información halladas. Esta plataforma facilitaría el monitoreo de la situación sanitaria del cáncer, desde diversos ángulos como el epidemiológico, económico, prestacional, entre otros. Este esfuerzo conjunto permitirá tener información organizada, focalizada y representativa del cáncer en el Perú y de esta manera poder contribuir a la ansiada atención integral, gratuita y digna que se merece la población afectada.
